# SPILLOVER EFFECTS OF A HUSBAND’S RETIREMENT ON A WOMAN’S HEALTH: EVIDENCE FROM URBAN CHINA

**DOI:** 10.1093/geroni/igz038.2246

**Published:** 2019-11-08

**Authors:** Emma Zang

**Affiliations:** Duke University, Durham, North Carolina, United States

## Abstract

This paper examines the effect that a man’s retirement has on his wife’s mental and physical health. I exploit the large increase in the probability of retirement at the legal retirement age for urban male wage earners in China as a natural experiment, using data from the China Health and Retirement Longitudinal Survey (CHARLS). I have implemented a fuzzy regression discontinuity design to compare the health outcomes of women whose husbands recently retired with those whose husbands are close to retire. My findings indicate that the retirement of her spouse improves physical and mental well-being of the woman, most likely by increasing the frequency of her social interactions and exercising. Although income and marital quality are less likely to be the main channels through which this positive spillover effect operates, decreased marital satisfaction or severe income constraints can be binding.

